# The Therapeutic Scope of Orofacial Mesenchymal Stem Cells

**DOI:** 10.3390/bioengineering12090970

**Published:** 2025-09-11

**Authors:** Bharath Chandra Vaddaram, Akhilesh Kumar Shakya, Brandon R. Zadeh, Diariza M. Lopez, Jon Wagner, Todd Parco, Umadevi Kandalam

**Affiliations:** 1 School of Biological and Health Systems Engineering, Arizona State University, Tempe, AZ 85281, USA; bharath.vbcr@asu.edu; 2 Department of Chemical Engineering, Texas Tech University, Lubbock, TX 79409, USA; akhilesh.shakya@ttu.edu; 3 Bioengineering, College of Engineering, Texas Tech University, Lubbock, TX 79409, USA; 4 Woody L. Hunt School of Dental Medicine, Texas Tech University Health Sciences Center, El Paso, TX 79905, USA; 5 L. Frederick Francis Graduate School of Biomedical Sciences, Texas Tech University Health Sciences Center, El Paso, TX 79905, USA

**Keywords:** oral mesenchymal stem cells, craniofacial tissue regeneration, autoimmune disorders, allergies, cartilage tissue regeneration, myocardium tissue regeneration, immunomodulation

## Abstract

Orofacial Mesenchymal Stem Cells (OMSCs) are an attractive and promising tool for tissue regeneration, with their potential for craniofacial bone repair being a primary focus of research. A key advantage driving their clinical interest is their accessibility from tissues that are often discarded, such as exfoliated deciduous teeth, which circumvents the ethical concerns and donor site morbidity associated with other stem cell sources. The high proliferation ability and multi-differentiation capacity of OMSCs make them a unique resource for tissue engineering. Recently, OMSCs have been explored in the restoration of the heart and skin, treatment of oral mucosal lesions, and regeneration of hard connective tissues such as cartilage. Beyond their direct regenerative capabilities, OMSCs possess potent immunomodulatory functions, enabling them to regulate the immune system in various inflammatory disorders through the secretion of cytokines. This review offers an in-depth update regarding the therapeutic possibilities of OMSCs, highlighting their roles in the regeneration of bone and various tissues, outlining their immunomodulatory capabilities, and examining the essential technologies necessary for their clinical application.

## 1. Introduction

Limitations in current craniofacial restorative methods have spurred interest in developing new approaches, such as stem cell-based regenerative therapies, a key component of regenerative tissue engineering. This field shows great promise as regenerative tissue engineering offers a great promise as it incorporates the use of the patient’s own cells rather than relying on artificial implant materials to replace the craniofacial bone. This engineering technique offers several potential benefits, including eliminating donor site morbidity, readily available cells, the absence of immune reactivity, and no risk of disease transmission [1,2]. To restore the damaged tissues, regenerative therapies utilize cells (especially stem cells), scaffolds (extracellular matrix), and growth factors (signals) or stimulants to promote their differentiation. Stem cells are undifferentiated cells that retain the capacity to self-renew and can differentiate into various cell types [3].

Mesenchymal Stem Cells (MSCs), first discovered in bone marrow by Friedenstein during the 1960s and 1970s, are a multipotent cell population capable of self-renewal and differentiation. For years, Bone Marrow Mesenchymal Stem Cells (BMSCs) have been used in research to generate various types of tissues, such as bone, cartilage, and other tissues [4]. The invasive and painful nature of bone marrow aspiration has led researchers to investigate alternative sources of MSCs. A growing body of evidence has legitimized the use of MSCs—derived from sources including the Hair Follicle Dermal Papilla/Skin-Derived MSCs [5], Synovial Membrane [6], and Umbilical Cord [7] to regenerate various tissues [4] such as adipose tissue [8,9,10,11], skin [5,12,13], cartilage [14], liver [15], lung [1], and orofacial tissue [5,16,17]. Among these, Orofacial Mesenchymal Stem Cells (OMSCs) have emerged as particularly promising candidates for craniofacial regeneration, largely due to their unique developmental origins [12,13,18]. The standard immunophenotypic profile for MSCs, established by the International Society for Cellular Therapy, is consistently observed in OMSCs. This includes the expression of key surface markers such as CD105, CD90, and CD73, coupled with the absence of hematopoietic and endothelial markers like CD45, CD34, CD14, and HLA-DR [19].

**Figure 1:**
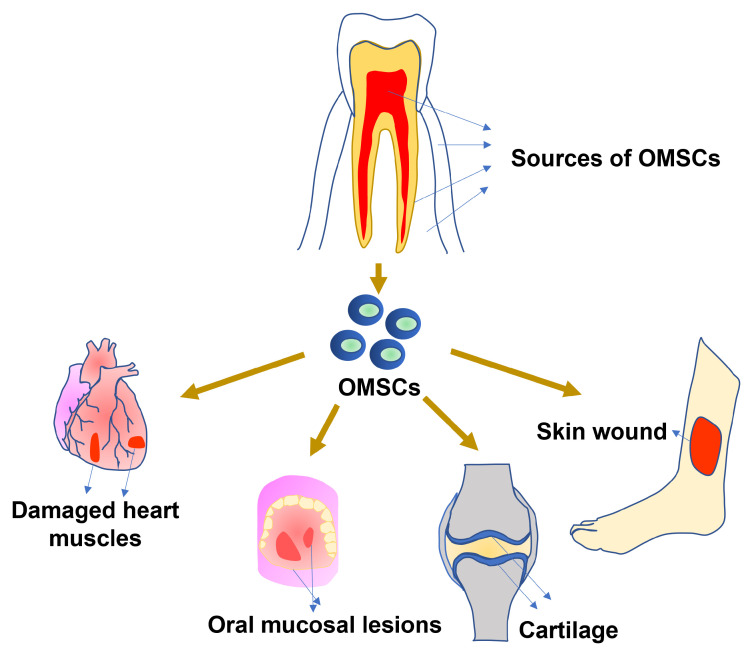
OMSCs’ role in the regeneration of different body tissues.

Many OMSCs originate from neural crest cells, which are formed during embryonic development through a process called neurulation [5,13]. Neurulation leads to the formation of the neural tube; cells that are not incorporated into the tube but arise during this process are known as neural crest cells [5]. Neural crest cells can migrate, giving rise to various tissues and cell populations throughout the adult body [5]. It has been demonstrated that neural crest cells remain throughout adulthood as MSCs, retaining their potential for self-renewal and the ability to differentiate into various cell lineages [5]. This shared developmental lineage likely confers a distinct regenerative advantage over MSCs from mesodermal sources, such as bone marrow [20]. This superiority is partly attributed to a principle of developmental tissue matching; since craniofacial bones are also of neural crest origin, using cells from the same embryonic lineage can lead to more effective and predictable healing compared to transplanting mesoderm-derived cells into a neural crest-derived environment [5,20,21]. This shared origin translates to a strong intrinsic potential for both osteogenic and soft tissue differentiation [11,20,21,22]. As a result, OMSCs often show improved differentiation potential towards craniofacial tissues and are regarded as superior candidates for neural regeneration, partly due to their increased secretion of essential neurotrophic factors compared to their mesodermal counterparts [5]. Research in recent years has demonstrated the ability of OMSCs to generate dental and bone tissue [18]. Due to their shared neural crest origin and their capacity to generate craniofacial tissues, OMSCs are highly appealing for research in craniofacial bone regeneration and immunomodulation in treating inflammatory disorders. OMSCs are attractive for craniofacial applications as they exhibit enhanced differentiation potential towards craniofacial tissues [12]. However, there are potential gaps in the orfacial stem cell-based research for clinical applications. The challenges include a lack of standardized protocols for isolation and differentiation due to their heterogeneity. A significant existing issue is the insufficient vascularization in created bone in engineered bone. Additionally, the mechanistic roles of these cells have yet to be fully understood. This review article examines the tissue regeneration capabilities of these cells and explores their differentiation potential and role in immunomodulation (Figure 1).

Several distinct types of OMSCs, each with unique therapeutic potential, have been isolated from orofacial tissue [12], and they are detailed in Table 1.

## 2. OMSCs in Craniofacial Bone Tissue Regeneration

While OMSCs hold therapeutic promise for a wide range of applications, its potential is most extensively studied in the context of skeletal repair [12,17]. In particular, functional reconstruction of bone defects in the craniofacial region, in particular, remains a significant clinical challenge. Traditionally, surgical reconstruction of bone tissue involves the implantation of autografts, allografts, xenografts, and guided bone regeneration using membranes [67,68,69].

### 2.1. Conventional Approaches to Bone Repair

Autogenous bone grafts, involves transplanting bone from a donor site to a deficient area within the same individual, are a common approach for repairing craniomaxillofacial defects. This method is considered the “gold standard” because it provides all three essential mechanisms for bone repair, osteoconduction, osteoinduction, and osteogenesis, and promotes excellent osteointegration [9,70]. A key advantage is the absence of an immunological reaction, with the patient’s iliac crest being a commonly used donor site. However, despite being the gold standard, limitations include donor site morbidity, a restricted supply of bone, and unpredictable resorption rates [71].

As an alternative, allografts use bone harvested from a different individual of the same species [72]. These grafts are processed to remove cellular components to minimize the risk of an immune reaction and disease transmission. Common examples of allograft include demineralized freeze-dried bone (DFDB), Demineralized Bone Matrix (DBM), and allogeneic freeze-dried bone (FDB). DFDB and DBM offer excellent osteoinductive properties and promote osteogenesis, while FDB is highly osteoconductive, thus providing a scaffold for new bone growth [73,74,75]. However, allografts carry risks, which include host incompatibility, disease transmission, infection, severe edema, bone resorption, and in some cases, premature sutural fusion or growth restriction [72,76,77]. Xenografts are derived from a different species and present another option, with bovine-derived particulate bone being the most common type [72,78]. Although this material can support bone augmentation and is gradually resorbed, clinical validation is limited, and further research is needed to confirm its efficacy [72,78]. A fourth approach, guided bone regeneration (GBR), is a surgical procedure that uses a barrier membrane to isolate a bone defect from the surrounding soft tissue [79]. This membrane restricts the influx of rapidly proliferating soft tissue cells, enabling osteoprogenitor cells and osteoblasts from the neighboring bone to settle at the site and create new bone in a controlled form [80,81]. However, a key limitation of GBR is the potential for membrane instability, resulting in suboptimal bone formation [79,82].

### 2.2. Tissue Engineering Strategies for Bone Regeneration

#### 2.2.1. The Role of Scaffolds

To overcome the limitations of conventional methods, tissue engineering strategies utilize biomaterial scaffolds to enhance bone regeneration. These are designed to mimic the natural extracellular matrix (ECM); these scaffolds serve as a delivery vehicle and supportive microenvironment for stem cells at the defect site [50]. Within this engineered microenvironment, a scaffold should fulfill several key functions: (1) retain and deliver cells, (2) support cell attachment, migration, proliferation, and differentiation into osteoblasts, (3) facilitate nutrient and waste diffusion, and (4) promote the integration and vascularization of the new tissue [82,83]. Critically for bone engineering, the scaffold must be osteoconductive, it must support the attachment of native bone cells and integrate with the host tissue.

Different materials have been used for this purpose, including ceramic-based scaffolds like Hydroxyapatite-Tricalcium Phosphate (HA-TCP) as well as biodegradable polymers such as Poly-lactic co-glycolic Acid (PLGA), which is a commonly preferred option [6,76,84,85,86]. Many studies using various materials have yielded good results; for example, Yamada et al. [87] used a Platelet-rich plasma (PRP) scaffold for a canine mandibular defect model, whereas Chamieh et al. [71] used a dense collagen scaffold for a rat calvarial defect model. Also, a comparative study by Nakajima et al. [88] investigated the use of Stem cells from exfoliated deciduous teeth (SHED) and compared their effectiveness to Dental Pulp Stem Cells (DPSCs) and BMSCs in healing calvarial bone defects using a PLGA scaffold. The results demonstrated that both SHED and DPSCs produced similar results regarding the regeneration of new bone. Notably, SHED exhibited a higher regeneration rate and a greater percentage of collagen and osteoid area. Another study by Putranti et al. [89] transplanted SHED with carbonate hydroxyapatite (CAP) granules using an atelocollagen sponge as a scaffold for treating surgically created artificial calvarial bone defects. A 3D analysis using microcomputed tomography, performed 12 weeks after transplantation, showed that the combination of SHED and CAP resulted in significantly greater bone regeneration than that in the other groups (Control, CAP, SHED). This was supported by histological and immunohistochemical evaluations showing enhanced expression of Bone morphogenetic protein 2 (BMP2), Vascular Endothelial Growth Factor (VEGF), and CD31. Furthermore, the study by Prahasanti et al. [90] compared a Hydroxyapatite (HA) scaffold alone with an HA scaffold combined with SHED for alveolar bone regeneration. Their findings indicated that the HA-SHED therapy significantly increased the expression of Osteoprotegerin (OPG) and decreased Receptor Activator of NF-Kb ligand (RANKL) expression, thereby modulating the OPG-RANKL system to favor bone formation. Finally Dai et al. [91] compared Periodontal Ligament Stem Cells (PDLSCs) seeded with scaffolds synthesized with chitosan (CS), β-glycerol phosphate (β-GP), and biphase calcium phosphate bone replacement material (HA/β-TCP) and concluded that a formulation of 2% CS, 12% β-GP, and 2% HA/β-TCP yielded favorable physical properties and biocompatibility both in vivo and in vitro conditions.

The physical design of scaffolds also plays a crucial role in their effectiveness. For instance, wavy polycaprolactone (PCL) scaffolds significantly enhance the osteogenesis of human MSCs compared to linear (orthogonal) designs. This improvement is reflected in higher levels of calcium deposition, increased alkaline phosphatase (ALP) activity, and more pronounced osteocalcin staining, which were confirmed through histological staining and quantitative biochemical assays for calcium deposition [92]. The enhanced osteogenesis is attributed to the curvature of the scaffolds, which promotes aligned and stretched cellular morphology with mature focal adhesions.

Although these findings are encouraging, it is essential to conduct thorough preclinical assessments using appropriate animal models to confirm their effectiveness and safety prior to clinical application. Evaluations comparing OMSCs and BMSCs have revealed a critical distinction between in vivo and in vitro outcomes. In vivo, OMSCs have demonstrated a bone regeneration capacity comparable to that of BMSCs [93,94]. However, in vitro studies present a more complex picture, with some evidence showing that BMSCs have superior properties, such as higher mineral deposition, proliferation rates, and expression of osteogenic markers, when compared to other dental-derived stem cells [7,95]. Therefore, while OMSCs are a strong candidate for in vivo regeneration, these subtle biological discrepancies warrant additional research to optimize their clinical application.

#### 2.2.2. Role of Growth Factors in Vascularized Bone

In addition to cells and scaffolds, growth factors are the third critical component of the tissue engineering triad. However, the function of growth factors goes beyond merely serving as osteoinductive substances that direct the differentiation of progenitor cells. Also, the long-term success of any engineered tissue construct fundamentally depends on its rapid integration with the host’s circulatory system, a process known as neovascularization [8,96]. Bone is a highly vascularized tissue, and a robust vascular network is essential for supplying oxygen and nutrients, removing metabolic waste, and providing conduits for immune cells and osteoprogenitors to access the regenerative site [97,98,99]. Because these cells are located beyond a diffusion limit of approximately 150–200 μm from a blood vessel, they are susceptible to necrosis, and insufficient vascularization is a primary cause of impaired fracture healing and bone graft failure [8,9,83,100,101].

Successful bone regeneration relies on a strong relationship between angiogenesis and osteogenesis, known as “angiogenic–osteogenic coupling.” This process involves the interdependence of new blood vessel formation and new bone development [96,98,100,102,103]. Central to this coupling mechanism is the interaction in which Bone morphogenetic proteins (BMPs) triggers the osteogenic process while also promoting the formation of the vascularature through VEGF. A recent study by Li et al. [104] fabricated cell constructs using both BMSCs and DPSCs, where DPSCs served as an inducer of vasculogenic factors and BMSCs as an osteogenic inducer. The immunofluorescence studies showed the co-localization of osteo- and angiogenic cells. The combination constructs with BMSCs/DPSCs showed a vascularized, scaffold-free organoid structure.

At the heart of this process are essential families of growth factors that regulate this interaction, especially BMPs and VEGF. BMPs are members of the Transforming Growth Factor-Beta (TGFβ) superfamily, the most powerful osteoinductive factors known, and capable of inducing osteoblastic differentiation from MSCs and promoting *de novo* bone formation [18,77]. Specifically, BMP2 and BMP-7 (also known as osteogenic protein-1 or OP-1) have been introduced clinically for treating bone defects [105,106,107]. Although they are mainly responsible for bone formation, their impact on the creation of new blood vessels is noteworthy. The angiogenic effects of BMPs are mostly indirect, mediated through their potent upregulation of VEGF expression in osteoprogenitor cells [72,76,98,108,109].

The formation of this vital vascular network involves two distinct processes. Angiogenesis refers to the process where new blood vessels sprout and grow from pre-existing vessels [8,103]. This is the more common method of new vessel formation in adults [110]. During angiogenesis, endothelial cells (ECs) are triggered by angiogenic growth factors to degrade their basement membrane, migrate into the tissue, proliferate, and form new vascular sprouts that eventually interconnect and stabilize into a functional network [8,102]. Vasculogenesis describes the formation of new blood vessels *de novo* (from scratch) [9,110]. This process involves the differentiation of *in situ* endothelial progenitor cells (EPCs) or angioblastic stem cells, and includes the migration of bone marrow-derived adult stem cells to the site of interest through systemic circulation, where they contribute to new vessel formation [9,98].

VEGF is arguably the most critical regulator of these processes, particularly angiogenesis in bone [83,97]. As a strong mitogen that specifically targets endothelial cells and its expression is influenced by physiological stimuli such as hypoxia (mediated by the master regulator HIF-1a) and various osteoinductive elements, including BMPs [102,103,109]. VEGF has multiple functions: it directly promotes the growth of endothelial cells and the formation of new blood vessels while also indirectly encouraging bone formation by stimulating endothelial cells to release osteogenic cytokines and by influencing the movement, growth, and development of osteoblasts [97,100,110]. However, the balance is delicate, so the overexpression of VEGF can lead to either aberrant vessel formation or even excessive bone resorption, thus highlighting the need for precise spatiotemporal control of these signals [83,98,103,111].

The intricate interactions among various signaling molecules that together facilitate the coupling of angiogenesis and osteogenesis are outlined in the Table 2. Modern tissue engineering approaches seek to utilize this biological interaction by developing bioactive settings that promote simultaneous bone development and blood vessel formation, frequently by incorporating cells, like OMSCs that secrete these critical factors [99,112].

In a study where combining pre-differentiated osteogenic Gingival Mesenchymal Stem Cells (GMSCs), a self-assembling hydrogel, and a low dose of BMP2 significantly accelerated alveolar bone regeneration, highlighting the power of combining a responsive cell type with targeted growth factor delivery [18]. Furthermore, OMSCs actively promote angiogenesis, a critical process for tissue viability and growth, through multiple mechanisms. They exert a strong paracrine effect by secreting higher levels of VEGF compared to other local cell types, thus stimulating host endothelial cell proliferation and the formation of new blood vessels [133]. Beyond signaling, these stem cells can also directly contribute to neovascularization by differentiating into functional endothelial cells that form vascular structures [134,135]. This endothelial differentiation is driven by VEGF signaling through the MEK1/ERK pathway, demonstrating a specific molecular mechanism for their vasculogenic potential [135]. The combination of these paracrine and direct differentiation capabilities allows OMSCs to effectively establish the vascular networks essential for supporting newly regenerated tissue [133,134].

To harness the therapeutic potential of these growth factors, significant research has focused on developing delivery systems that provide spatiotemporal control, aiming to mimic the natural healing cascade. The most common approach involves incorporating growth factors into biomaterial scaffolds, such as hydrogels, ceramics, and synthetic polymers, which provide localized and sustained release as the material biodegrades [82,136]. More advanced strategies include cell-based delivery, in which stem cells are genetically modified to secrete specific growth factors, and gene therapy using viral vectors to ensure prolonged expression at the defect site [6,8]. A key strategy for spatiotemporal control is the sequential delivery of factors; for instance, releasing an angiogenic factor like VEGF first to establish a vascular network, followed by an osteogenic factor like BMP2 to promote bone formation [137,138,139].

Even with these advancements, there are significant challenges to achieving clinical translatability. Although rhBMP-2 and rhBMP-7 are clinically approved for specific applications, their efficacy can be unpredictable, with human trials showing a lower regenerative response than promising preclinical animal studies [77,82,140,141]. A primary challenge is dosage control; supraphysiological doses can lead to adverse effects, including inflammation, edema, aberrant vessel formation, and concerns regarding tumorigenicity [77,98,99]. The short in vivo half-life of these proteins further necessitates sophisticated delivery vehicles [99,112]. Therefore, while the principles are well-established, optimizing the safety, dosage, and release kinetics of these powerful biological agents remains a critical barrier to their widespread and predictable clinical use.

#### 2.2.3. Preclinical Evaluation of Bone Reconstruction in Animal Models

The effectiveness of therapies utilizing OMSCs is thoroughly evaluated in preclinical animal models, which are crucial for confirming both the safety and regenerative capabilities of novel constructs prior to their clinical translation. Researchers have utilized various defect models to simulate clinical scenarios, most commonly creating critical-size defects in the calvaria, mandible, and alveolar ridge of animals like rats and canines. Direct comparisons of OMSC-seeded scaffolds against the gold standard BMSC-seeded scaffolds under identical experimental conditions have yielded varied but informative results [43,93]. These studies are essential for evaluating the performance of various types of OMSCs types against each other, against traditional sources like bone marrow, and against the clinical gold standard of autologous bone grafting.

Recent studies provide compelling, albeit sometimes conflicting, evidence for the potent osteogenic capacity of OMSCs. For example, a comparative study by Yamada et al. [87] directly evaluated Bone Marrow MSCs (cMSCs), adult Dental Pulp Stem Cells (cDPSCs), and deciduous tooth stem cells (pDTSCs) in a canine mandibular defect model. They found that all three stem cell types, when delivered in combination with PRP scaffold, produced robust and equivalent amounts of new bone, significantly outperforming PRP-only and empty defect controls. Another study by Jahanbin et al. [142] tested the efficacy of DPSCs, pre-differentiated into osteoblasts, for repairing maxillary alveolar defects in rats. Their findings demonstrated that after two months, the DPSC-scaffold construct achieved a degree of bone formation comparable to that of an autologous iliac bone graft, the current clinical gold standard. However, while most studies validate these outcomes in preclinical settings, none have yet reported on the long-term feasibility of OMSC-mediated bone remodeling, including comprehensive validation of vascularization and biomechanical strength. Additionally, Chamieh et al. [71] demonstrated that introducing rat DPSCs into a dense collagen gel scaffold notably enhanced bone regeneration in a rat calvarial defect model when compared to utilizing the acellular scaffold alone. A comparison of different animal defect studies, which are designed to mimic acute injury, is presented in Table 3.

However, while these preclinical outcomes are promising, they also highlight significant challenges that temper enthusiasm for immediate clinical translation. A critical issue is the frequent observation of incomplete bone regeneration, where the engineered construct fails to fully bridge the defect and, in some cases, provides no evidence of enhanced bone formation in non-critical-sized defects. This can be exacerbated by suboptimal scaffold properties; for instance, a scaffold that does not degrade at a rate commensurate with new tissue formation can physically obstruct healing and entrap the delivered cells, as was observed in a study using a hyaluronic-based hydrogel [149]. Other cell types have shown promise, such as Buccal Fat Pad-Derived Stem Cells (BFPDs), which improved bone regeneration and mineral density in both healthy and osteoporotic rats when cultured on bioceramics. Furthermore, the inherent complexity of craniofacial anatomy and the specialized nature of its tissues present a high bar for success [12]. The success of regeneration is also profoundly influenced by the embryonic origin of the transplanted cells. A mismatch between neural crest-derived Alveolar Bone-Derived Mesenchymal Stem Cells (ABMSCs) and the host site can disrupt healing, highlighting that stem cells from different skeletal locations are not always interchangeable [20]. These findings emphasize that despite positive results, a deeper understanding of the in vivo mechanisms after transplantation is necessary, along with the development of more advanced scaffolds that can better orchestrate the complex interplay of cellular and matrix events in bone repair [57,82].

Taken together, the evidence from these preclinical models solidifies the role of OMSCs as a premier cell source for craniofacial bone repair. The enhanced performance of OMSCs in these craniofacial models is not attributed to a single characteristic but rather a combination of faster proliferation, potent immunomodulatory functions, and, most critically, cell-intrinsic properties derived from their neural crest origin [150,151]. Many OMSCs populations exhibit significantly higher proliferation rates compared to traditional sources like BMSCs, allowing for more rapid expansion of therapeutically relevant cell numbers [13,94,151]. Furthermore, their ability to modulate the host immune response helps create a pro-regenerative environment conducive to healing [152]. However, their most significant advantage may be their embryonic lineage. This shared neural crest origin with most craniofacial bone and nerve tissue provides a “developmental matching” that makes them uniquely suited for regenerating these specific structures, a phenomenon not observed with mesoderm-derived MSCs [150]. These studies collectively show that stem cells derived from dental pulp and exfoliated deciduous teeth can rival and, in some contexts, exceed the potential of traditional sources like BMSCs [87]. Their demonstrated ability to generate bone comparable to autologous grafts, coupled with their high proliferation rates and relative ease of access from discarded tissue, makes them an exceptionally attractive tool for oral surgery and orthopedics [71,142]. Although bone regeneration is the most researched area, the distinctive biological characteristics of OMSCs, including their ability to differentiate into multiple cell types and their origin from the neural crest, indicate a much wider range of therapeutic possibilities. The following sections will explore the exciting potential of these versatile cells in regenerating various soft tissues and modulating complex immune responses.

## 3. OMSC for Other Tissue Regeneration

### 3.1. Regeneration of Soft Tissues

#### 3.1.1. Cartilage Tissue

Similar to bone, repairing cartilage defects caused by trauma or degenerative conditions like Osteoarthritis represents a major clinical challenge [6,26]. This difficulty is largely due to the fact that cartilage is an avascular and aneural tissue, giving it a very limited capacity for self-repair. While current restorative techniques such as autologous chondrocyte transplantation exist, there remains a high demand for improved methods that can regenerate native articular cartilage with its full biological and mechanical properties [26,112]. To address this, tissue engineering strategies utilizing OMSCs have shown significant promise. A key study by Moshaverinia et al. [153] explored the potential of PDLSCs and GMSCs for regenerating cartilage in the temporomandibular joint (TMJ) disc, a site of common cartilage injury in the craniofacial complex. These particular OMSCs were chosen for their easy accessibility from tissues often discarded during dental procedures. In the study, the cells were seeded onto a Arginine-Glycine-Aspartic Acid tripeptide (RGD)-coupled alginate hydrogel, which was found to successfully support their chondrogenic differentiation and metabolic activity both in vitro and in vivo conditions. Both PDLSCs and GMSCs generated a considerably greater quantity of colony-forming clusters in comparison to conventional BMSCs. Another study by Mata et al. [154] demonstrated the chondrogenic potential of DPSCs cultured in 3% alginate hydrogels for both in vitro and in vivo conditions; three months post-surgery, the animals implanted with DPSCs exhibited notable cartilage regeneration.. This led to the successful generation of new cartilage tissue in the animal model, which was then compared to native cartilage based on histological and morphological characteristics. The findings suggest that stem cells from dental sources can be efficiently used for cartilage tissue engineering when paired with an appropriate hydrogel carrier [95].

#### 3.1.2. Cardiac Tissue

Ischemic heart diseases, such as myocardial infarction (MI), are a leading cause of mortality. Following an MI, damaged cardiac tissue is replaced by non-functional fibrous scar tissue, impairing heart function [101,155,156,157]. Due to the significant limitations of traditional treatments like organ transplantation, research has focused on regenerative strategies, including the use of OMSCs like DPSCs [25,101].

The therapeutic potential of DPSCs is largely attributed to their paracrine activity. They secrete a variety of cardioprotective cytokines and growth factors, including VEGF, Insulin-like Growth Factors (IGF-1, IGF-2), Stem Cell Factor (SCF), and Granulocyte-Colony Stimulating Factor (G-CSF), which collectively exert pro-angiogenic and anti-apoptotic effects on damaged heart tissue [158,159]. A key preclinical study validated this by inducing MIs in rats and treating them with DPSCs seven days later. The DPSC-treated group showed significant improvements in systolic function, increased blood vessel density (angiogenesis), and reduced fibrous scar formation compared to controls [158]. Unfortunately, their long-term therapeutic capability has not been explored yet.

Further evidence for the efficacy of dental stem cells comes from studies on SHED. In a mouse model of myocardial injury, a CM from SHED (SHED-CM) reduced the myocardial infarct size, decreased apoptosis, and lowered levels of inflammatory cytokines such as TNF-α, IL-6, and IL-1β in comparison to the conventional Dulbecco’s Modified Eagle’s Medium (DMEM) [37]. The study by Yamaguchi et al. [37] demonstrated that the therapeutic outcomes from this SHED-CM were superior compared to those from the standard DMEM media control. In vitro, SHED-CM also protected cultured cardiac myocytes from hypoxia-induced apoptosis. This potent anti-apoptotic effect was attributed to a higher concentration of Hepatocyte Growth Factor (HGF) in SHED-CM compared to medium from bone marrow or adipose-derived stem cells [37].

Collectively, these findings underscore the potential of DPSCs and SHED-based therapies for managing ischemic heart disease by promoting neovascularization, reducing inflammation and apoptosis, and limiting adverse cardiac remodeling. However, no studies have yet reported long-term therapeutic effects of DPSCs. Nonetheless, other types of stem cells, such as BMSCs, have shown sustained improvements in left ventricular ejection fraction at 6-, 12-, 24-, and 36-month post-transplantation [160,161,162].

#### 3.1.3. Muscle Tissue

While skeletal muscle can regenerate from minor injuries via satellite cells, this process fails after severe trauma, leading to myofiber atrophy and fibrous scar tissue [163,164,165]. MSCs are a promising therapy due to their regenerative and immunomodulatory properties [165]. MSCs treatment promotes the long-term maintenance of regenerated myofibers, can favor the formation of fatigue-resistant fibers, and balances the regenerative environment [163]. Additionally, extracellular vesicles (EVs) derived from MSCs can promote muscle and blood vessel growth while decreasing fibrosis [166]. The therapeutic potential of MSCs is influenced by their tissue origin, making cell source selection a critical factor.

OMSCs possess a reasonable capacity for myogenic differentiation. For instance, Zhang et al. [167] demonstrated that treating DPSCs with Noggin, an antagonist of BMPs, promotes the formation of myotubes. By regulating BMP/Smad signaling, Noggin can accelerate the generation of satellite-like cells from DPSCs, which may possess a rapid capacity for muscle regeneration. Complementing these findings, an in vivo study by Martínez-Sarrà et al. [168] showed that when DPSCs were engrafted into dystrophic mouse models, the cells successfully integrated into both muscle fibers and associated blood vessels.

#### 3.1.4. Retinal and Neural Regeneration

The unique neural crest origin of OMSCs makes them particularly compelling candidates for neural and retinal regeneration, a frontier extending beyond their well-established applications in musculoskeletal repair [5,13,25,50,71]. Among the most studied are DPSCs [13,25,71,83] and SHED [57,71,83], which exhibit remarkable neurogenic potential. These cells can differentiate into various neural lineages, including neuron-like cells and oligodendrocytes, and possess powerful neuroprotective effects through a paracrine mechanism, secreting a cocktail of critical neurotrophic factors like Brain-derived neurotrophic factor (BDNF) and Nerve growth factor (NGF) that support nerve survival and growth [25,30,41,50,65]. In preclinical models of spinal cord injury, Nosrat et al. [169] found that DPSCs promote locomotor recovery by reducing inflammation and supporting axon regeneration [169]. The ability to protect the nervous system also applies to the visual system, where these cells or the factors they release have demonstrated effectiveness in safeguarding retinal ganglion cells following optic nerve damage and reducing neuroinflammation, which in turn enhances visual performance and slows down the degeneration of photoreceptors [41,57]. In some in vitro studies, dental neurospheres have been subjected to electrophysiological measurements, such as intracellular calcium imaging and patch clamp recordings, to confirm normal neural function; these measurements primarily assess general neural activity rather than specific visual function in an in vivo context. They also pointed out the significant lack of in vivo studies using dental spheres for treating neurodegenerative diseases and called for further electrophysiological investigations to explore neural physiological functions, particularly in experimental outcomes conducted in vivo [65,170,171,172].

This potential is not limited to dental pulp-derived cells. Other easily accessible sources, including PDLSCs, DFSCs, and GMSCs, also share this inherent capacity for neural repair, demonstrating the ability to differentiate into Schwann-like cells and other neural progenitors [43]. For example, a study by Zhang et al. [43], GMSCs were successfully used to promote facial nerve regeneration in rat models, especially when delivered via advanced 3D-printed nerve conduits. Another study by Ansari et al. [173] showed that PDLSCs and GMSCs encapsulated in a 3D scaffold based on alginate and hyaluronic acid hydrogels sustained the release of human nerve growth factor (NGF) and expressed high levels of neurogenic differentiation genes via qPCR. In a study, Stem Cells from Apical Papilla (SCAPs) spheres showed increased expression of MAP-2 and NeuN, while Nestin and Musashi-1 (immature neural markers) decreased [174]. Furthermore, a study used CM from SHED-CM aids recovery in cerebral ischemia models by reducing infarct volume and promoting neurogenesis and vasculogenesis [37]. These therapeutic effects are comparable to those achieved with the direct transplantation of DPSCs [175]. The potential of orofacial stem cells to differentiate into dopaminergic neurons and provide neuroprotective effects makes them promising candidates for treating diseases such as Parkinson’s and Alzheimer’s. In animal models of Parkinson’s disease, transplanted SHED have differentiated into dopamine-producing neurons, leading to improvements in behavioral deficits [176]. In in vitro models of Alzheimer’s disease, DPSCs have been shown to reduce amyloid-beta-induced toxicity and enhance neuronal viability. The diverse family of OMSCs offers significant advantages for neural and retinal regeneration due to their combined ability for neurogenic differentiation, their secretion of crucial neurotrophic and immunomodulatory factors, and the ease of harvesting these cells [5,13,41,150]. While their therapeutic potential is often enhanced when combined with scaffolds to improve cell delivery, further research is necessary to fully optimize their clinical application and understand long-term outcomes [17,177].

#### 3.1.5. Dentin-Pulp Complex

In addition to bone and cartilage, OMSCs play a crucial role in initiatives aimed at regenerating the dentin-pulp complex [25]. This process is heavily modulated by growth factors like PDGF, which not only contribute to general wound healing but also specifically promote the proliferation of cultured DPSCs and the synthesis of dentin matrix proteins, while inhibiting premature alkaline phosphatase (ALP) activity [159]. Similarly, members of the TGF superfamily, particularly BMP2, are potent inducers of odontoblastic differentiation. Both in vitro and in vivo studies have shown that BMP2 effectively guides DPSCs and SCAPs to differentiate and form new odontoblasts, the cells responsible for producing dentin [57,159].

### 3.2. Immunomodulatory Functions of OMSCs

**Figure 2:**
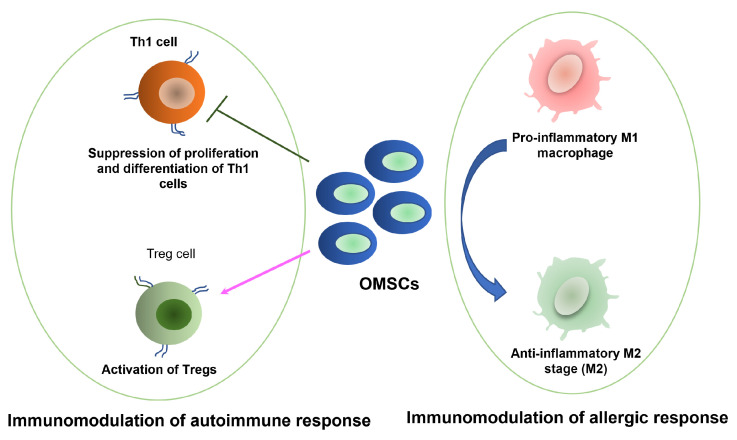
OMSCs mediated immunomodulation.

Beyond their direct regenerative capacity, OMSCs possess potent immunomodulatory properties that are increasingly being harnessed to treat a spectrum of inflammatory and autoimmune disorders [41]. This therapeutic action is largely mediated by their secretome—a rich milieu of cytokines, chemokines, and growth factors—which allows them to steer aberrant immune responses back toward a state of homeostasis [1]. This capability, combined with their accessibility from discarded tissues, positions OMSCs as a highly promising platform for cellular immunotherapy [178]. Preclinical studies have begun to elucidate the specific mechanisms by which these cells quell inflammation and restore immune tolerance, as illustrated in Figure 2.

#### 3.2.1. Application in Wound Healing, Allergy, and Inflammation

The immunomodulatory prowess of OMSCs is clearly demonstrated in their ability to influence wound healing and mitigate inflammatory and allergic responses by acting on key cells of both the innate and adaptive immune systems.

A prime example is in cutaneous wound healing, an extremely complex process where an uncharacteristic inflammatory response can lead to scar formation and fibrotic disease [44]. Macrophages are critical players in all phases of healing and can be polarized to a pro-inflammatory (M1) or a pro-resolving (M2) state [44]. Leveraging their potent immunomodulatory properties, GMSCs can reprogram pro-inflammatory M1 macrophages into the pro-resolving M2 phenotype, leading to improved tissue remodeling. For instance, a study by Hong et al. [179] demonstrated that co-culturing GMSCs with macrophages significantly reduced the expression of M1 markers (TNF-α, IL-6, IL-1β, CD86, and HLA-DR) while moderately increasing M2 markers like IL-10 and CD206 [179]. This reprogramming resulted in a significant increase in the expression of the mannose receptor (MR; CD206), a well-accepted marker for M2 macrophages, as determined by flow cytometric analysis. Crucially, these benefits extend to preclinical models without requiring host immunosuppression. Human GMSCs systemically administered into immunocompetent mice were shown to migrate to skin wound beds, accelerate wound closure, promote re-epithelialization and angiogenesis, and suppress local inflammatory cell responses [44]. Further supporting their potent immunomodulatory capacity, systemic administration of human GMSCs had stronger beneficial effects than BMSCs in prolonging skin allograft survival and delaying allograft rejection [180]. The use of 3D spheroid culture can further enhance these effects, as spheroid GMSCs show increased resistance to oxidative stress and enhanced homing ability [13,181]. In Zhang et al. [181], an in vivo murine model of chemotherapy-induced oral mucositis demonstrated that spheroid-derived GMSCs had greater therapeutic efficacy than adherent GMSCs in reversing body weight loss and promoting regeneration of the disrupted epithelial lining in mucositis tongues. These findings suggest that 3D spheroid culture helps preserve early stemness and may precondition GMSCs for enhanced treatment of oral mucositis.

This mechanism of macrophage polarization extends to other inflammatory conditions. The clinical need for novel anti-inflammatory therapies is particularly evident in conditions like severe asthma, which remains poorly controlled by conventional treatments [182]. The therapeutic potential of OMSCs has been demonstrated in relevant preclinical models; for example, CM from SHED was shown to induce anti-inflammatory M2-like macrophages and attenuate lung injury in a mouse model of the condition [183]. Similarly, DFSCs can facilitate the conversion of pro-inflammatory M1 macrophages to the M2 phenotype, also determined by flow cytometric analysis [178]. This cellular shift is accompanied by a significant change in the cytokine environment: the levels of pro-inflammatory cytokines such as TNF-α, IL-1, and IL-6 are downregulated, while the concentration of the anti-inflammatory cytokine IL-10 is increased [178,179]. This finding is critical because it shows that OMSCs can fundamentally alter the inflammatory milieu at a cellular level, promoting a switch from a destructive to a regenerative environment.

In addition to modulating the innate immune system, OMSCs also powerfully regulate the adaptive immune response, which is central to the pathology of allergic diseases. Pathologically, asthma is characterized as a T-helper 2 (TH2) cell-mediated inflammatory disease that results in chronic airway inflammation and tissue remodeling [184]. Investigating this connection, an in vitro study by Genç et al. [185] and Genç et al. [186] demonstrated that when co-cultured with peripheral blood mononuclear cells (PBMCs) isolated from asthmatic patients and cultured under in vitro conditions. The results showed that DFSCs effectively suppressed the proliferation of activated CD4^+^ T-lymphocytes in culture conditions, the key drivers of the TH2 response [185]. Furthermore, this immunosuppressive effect could be significantly enhanced by pre-treating the DFSCs with interferon-gamma (IFN-γ), indicating that the cells’ therapeutic activity efficacy can be a future cell-based treatment for allergic diseases like asthma [185,186]. The dual capacity to polarize macrophages and suppress T-cell activation makes OMSCs a uniquely versatile tool for treating complex inflammatory disorders.

The immunomodulatory mechanisms of OMSCs involve both paracrine signaling and direct cell–cell contact. The secretory mechanism of DFSCs involves the release of factors such as TGF-β, which in turn modulate the immune response by reducing pro-inflammatory cytokines, like upregulating Treg-associated IL-10 [51,178,187]. A limitation of the research on specific T-cell responses in asthma is that findings are primarily derived from in vitro culture conditions [185,188]. Consequently, no in vivo studies have yet been performed to determine if systemic versus local delivery of these cells leads to differential T-cell suppression. Nevertheless, the dual capacity to polarize macrophages and suppress T-cell activation makes OMSCs a uniquely versatile therapeutic tool for complex inflammatory disorders.

#### 3.2.2. Application in Autoimmune Disorders

Autoimmune disorders, which represent a leading cause of mortality and morbidity, arise when the immune system mistakenly attacks the body’s own tissues [189]. Current treatments primarily rely on broad immunosuppressive drugs that, while effective, can leave patients vulnerable to infections and other serious side effects. OMSCs, prized for their profound immunomodulatory functions and ease of acquisition from sources like the dental follicle, offer a paradigm-shifting approach that favors targeted immune tolerance over general suppression [189]. One study established a co-culture system of human peripheral blood-derived lymphocytes and OMSCs to investigate immunomodulatory effects. It found that Human Olfactory-MSCs (Of-MSCs) effectively suppressed the CD4+IFN-γ+ T cell proportion in vitro [152]. This suggests “biological crosstalk” between Of-MSCs and host cells, both in co-culture models and in vivo via paracrine readouts of Serum Cytokine, Immune Cell Proportions, and Signaling Pathway Activity [152].

The therapeutic potential of OMSCs has been demonstrated in preclinical models of specific autoimmune diseases, such as Myasthenia Gravis (MG). MG is an antibody-mediated autoimmune condition where pathogenic antibodies attack the neuromuscular junction, leading to profound muscle weakness. In a mouse model of experimental autoimmune myasthenia gravis (EAMG), treatment with DFSCs led to remarkable clinical improvements [52]. The study by Ulusoy et al. [52] reported that DFSC-treated mice exhibited less muscle weakness, had significantly low serum levels of the disease-causing anti-MuSK antibodies, and showed reduced deposition of immune complexes (IgG and C3) at the neuromuscular junction. This was accompanied by decreased lymph node cell proliferation and lower systemic levels of inflammatory cytokines like IL-6 and IL-12. These findings provide compelling evidence that OMSCs can intervene at multiple levels of the autoimmune cascade. To date, no studies have investigated the long-term or rebound effects of OMSC-mediated immunotherapy in autoimmune diseases. Exploring these aspects could provide valuable insights into the sustained therapeutic potential of OMSCs.

Furthermore, OMSCs have shown efficacy in T-cell-driven autoimmune conditions like Rheumatoid arthritis (RA), a systemic disease characterized by chronic joint inflammation. A key defect in RA is the dysfunction of Treg, a specialized immune cell population responsible for maintaining self-tolerance. A study by Zibandeh [188] explored the interaction between DFSCs and PBMCs from RA patients. The results revealed that DFSCs were able to suppress the proliferation of aggressive, disease-causing lymphocytes and, critically, significantly increase the frequency of protective CD4^+^CD25^+^Foxp3^+^ Treg cells. This effect has also been observed in models of Amyotrophic Lateral Sclerosis (ALS), where DFSCs also increased the Treg cell ratio in lymphocytes of patients [48]. While FoxP3 staining confirmed the increase in Tregs, the specific role of cytokines like IL-2 or IL-10 in this process was not detailed in the study. By bolstering the Treg population, DFSCs help restore the immune system’s natural braking mechanism, a highly targeted strategy to control autoimmunity. The ability of OMSCs to effectively modulate distinct pathological mechanisms—be it antibody-mediated damage in experimental autoimmune myasthenia gravis (EAMG) [52] or T-cell dysregulation in RA under in vitro conditions via secretions of cytokines like IL-10 secreting T cells [188].

The demonstrated versatility of OMSCs, from rebuilding musculoskeletal tissues to orchestrating complex immune responses, positions them as a powerful therapeutic modality for a wide range of clinical challenges [25,51]. However, translating this profound biological potential from preclinical models to routine clinical practice is not without significant hurdles. Key challenges remain, including the effective delivery and retention of cells at the target site, the creation of functional vascular networks within large tissue constructs, and the need to overcome the inherent complexities of directing cell fate in vivo [39,83]. The successful clinical translation of OMSC-based therapies will therefore depend critically on the convergence of cell biology with advanced bioengineering strategies. The next section explores the enabling technologies—such as 3D bioprinting, artificial intelligence, and microfluidics—that are being developed to overcome these barriers and accelerate the development of personalized regenerative solutions.

## 4. Challenges, Limitations, and Reasons for Conflicting Data

While OMSCs present a promising avenue for regenerative medicine, their journey toward widespread clinical adoption is constrained by significant hurdles. The “translational gap” between promising preclinical results and consistent clinical application is largely due to a pervasive lack of standardization and the inherent complexity of biological systems. A clear understanding of these constraints is essential for guiding future research and development.

### 4.1. Intrinsic Biological Constraints

A primary constraint is the limited and biased differentiation capacity of OMSCs. Like all MSCs, they are multipotent, not pluripotent, meaning their differentiation is largely confined to mesenchymal lineages such as bone, cartilage, and fat, and they cannot form all cell types of the body [11,101,190]. This inherently restricts their use in regenerating complex organs that rely on precise epithelial–mesenchymal interactions [11]. Furthermore, the distinct embryonic origins of craniofacial bones (neural crest) versus the appendicular skeleton (mesoderm) create functional differences between OMSCs populations and those from other sites [11,191,192,193]. A mismatch in embryonic origin between transplanted cells and the host bone can disrupt regeneration; for example, mesoderm-derived iliac crest BMSCs may differentiate into chondrocytes instead of osteoblasts when transplanted into a neural crest-derived mandibular defect [20,194,195]. This indicates that stem cells from different skeletal locations are not always interchangeable, making OMSCs potentially less effective for non-craniofacial tissues despite their superiority for craniofacial bone repair [6,18]. This lineage bias is also evident among specific OMSC subtypes: ABMSCs show poor chondrogenic and adipogenic potential compared to iliac BMSCs, and GMSCs often have weak osteogenic capacity, potentially due to inactivity within certain protein kinase pathways [53,55,196].

Beyond lineage bias, the process of preparing cells for therapeutic use presents its own challenges. The progressive subculturing required to expand cell numbers can negatively alter cellular phenotypes, as prolonged two-dimensional (2D) culture and serial passages may lead to a loss of self-renewal capacity and, ultimately, senescence [65]. While some sources suggest that GMSCs maintain stable morphology at higher passage numbers [94], this remains a general challenge for most MSCs. Moreover, MSCs in general exhibit limitations in replicating the full complexity of native tissues. For instance, they struggle to differentiate into all the functional, terminally differentiated cells, such as osteocytes and osteoclasts, which are essential for the intricate, life-long processes of bone remodeling [197]. Even when OMSCs show potential for non-mesenchymal lineages like neurogenesis, they often fail to differentiate into fully functional neurons in vivo or become skeletal myofibers in muscle regeneration [41,93,165,166,198].

### 4.2. Methodological and Translational Challenges

The transition from the laboratory to the clinic is further hampered by logistical issues and unpredictable cell behavior in vivo. OMSCs are often available only in limited quantities, with low cell yields from orofacial bone marrow aspirates and dental tissues, making it difficult to regenerate large defects [11,17]. This necessitates extensive in vitro expansion, a laborious and expensive process that can reduce cell potency, compromise differentiation ability, and increase the risk of senescence or malignant transformation.

To address this, strategies like 3D spheroid culture are being explored. By allowing tight cell–cell and cell–matrix interactions that mimic the in vivo microenvironment, spheroid culture has been shown to enhance the stemness, multipotency, and secretome profiles of MSCs, while also improving their resistance to oxidative stress. Another key strategy is the development of improved, serum-free media formulations [13,199,200,201]. Traditional reliance on fetal bovine serum (FBS) carries risks of disease transmission and immunogenic reactions. The use of serum-free media or humanized substitutes like platelet lysate (PL) improves reproducibility and clinical safety [39,76,164].

Once transplanted, MSCs frequently exhibit poor engraftment and survival, as their fate is significantly influenced by the harsh, ischemic, or inflammatory microenvironment of the recipient niche [26,57]. The clinical application of cell-based tissue engineering in craniofacial bone is still very limited, with few studies utilizing promising sources like BFPDs, highlighting a need for more randomized controlled trials [57,82,202,203]. A significant ambiguity remains regarding whether the observed benefits result from donor cell differentiation or from paracrine-mediated activation of host repair mechanisms. This obscures the true mechanism of action, making it challenging to optimize therapies and predict outcomes [2,17,93,149,152,181]. Recently, the field has started to close this translational gap, with several registered clinical trials evaluating the safety and efficacy of specific populations of OMSCs, particularly DPSCs, in human patients. For bone and periodontal regeneration, notable trials like NCT03386877 [204] have provided Level 1 evidence demonstrating that DPSC-based therapies can significantly improve bone fill and clinical attachment in chronic periodontitis. In the realm of neural disorders, the J-REPAIR trial (NCT04608838) [205] represents a landmark study investigating an allogeneic DPSCs product for acute ischemic stroke, signaling a move towards “off-the-shelf” cell therapies. While trials for cardiac disorders are still forthcoming, these groundbreaking studies in bone and neural repair are starting to translate the extensive preclinical promise of OMSCs into validated clinical applications. Preclinical studies are summarized in the Table 4.

### 4.3. Potential Reasons for Conflicting Data

Conflicting or inconsistent results across studies are a common challenge, stemming from several key differences in experimental design and execution.

#### 4.3.1. Animal Models

Animal models, particularly rodents, often yield unreliable results due to fundamental differences in physiology and immune systems compared to humans [190,212]. The regenerative reaction to rhBMP-2, for example, was significantly reduced in humans compared to findings from animal studies [6]. Furthermore, models often use young, healthy animals, which do not reflect the older, comorbid patient populations seen in clinics [102,108]. The use of immunocompromised animals, while necessary for xenografts, fails to account for the host immune response [213].

#### 4.3.2. Cell Isolation and Culture Techniques

MSCs are inherently heterogeneous [2,13]. Their properties vary significantly based on tissue source, donor age, and, critically, the isolation and expansion protocols used [2,8,56,77,202,214]. Different enzymes and techniques (explant vs. enzymatic digestion) can yield different subpopulations [22,164,196]. Long-term culture and high passage numbers are known to alter cell morphology and reduce differentiation potential [13]. Variations in culture media (e.g., serum vs. serum-free) also contribute to variability [215]. For example, the stability of the immunophenotypic profile can depend on both the donor and culture conditions. A study comparing healthy and periodontitis-affected DPSCs showed no difference in CD73, CD90, or CD105 at early passage stages. In contrast, in later passages, DPSCs obtained from periodontitis-affected tissue showed upregulated HLA-DR expression [216]. In another study, Qu et al. [217]. demonstrated significant downregulation of CD105 expression in the sixth passage of DPSCs compared to the second passage. Taken together, these studies have shown that the immunophenotypic profile depends on both the donor and the culture conditions. Additionally, recent proteomic studies have started to identify the molecular differences that underlie the functional diversity among sources of OMSCs. Earlier research on the secretome showed that SHED expresses more immunomodulatory and osteogenic cytokines, while DPSCs produces a greater amount of angiogenic factors. A thorough analysis of the intracellular proteome offers a deeper understanding of their unique molecular machinery [218]. Using data-independent acquisition proteomics, a recent study identified 209 differentially expressed proteins between the two cell types [218]. The analysis revealed that DPSCs are characterized by higher expression of proteins involved in mitochondrial energy metabolism (e.g., NDUFB family), autophagy (RPTOR), and innate immunity (TLR3) [218]. In contrast, SHEDs showed elevated levels of proteins associated with glycerolipid metabolism and a greater potential for adipogenic differentiation (e.g., AKR1B family). These distinct proteomic signatures highlight fundamental differences in the metabolic and regulatory states of these cell populations, which likely contribute to their varied therapeutic potentials and may explain inconsistencies in preclinical data. Moreover, the choice of immunophenotype markers utilized for characterization influences the expression of these markers. Research has shown that the age and health status of the donor have an impact on the expression of these immunophenotype markers [216]. Hence, it is essential to develop a fundamental set of criteria specifically designed for OMSCs.

#### 4.3.3. Scaffold Properties and Delivery Systems

The wide variety of materials (such as PCL, collagen, and HA) makes direct comparison challenging [96,219]. Optimizing key properties such as degradation rate and porosity is challenging due to conflicting data on the ideal parameters for bone formation and vascularization [6,109,220]. This is compounded by a lack of standardization for biological augments like PRP and Platelet-rich fibrin (PRF), leading to inconsistent clinical outcomes [11,57,219,221]. Furthermore, many commercial biomaterials lack comprehensive characterization [79].

The delivery of growth factors has also been thoroughly studied in the context of stem cell-based regenerative technologies. While microencapsulation in hydrogels for sustained release is a widely used method, gene therapy and the synergistic use of two growth factors are also employed. Although preclinical studies have been successful, a significant challenge remains in clinical use due to their supraphysiological doses and short half-life. More studies have to be evaluated for their clinical use [222,223].

#### 4.3.4. Exosome Preparation

A lack of standardized protocols for the extraction, purification, and storage of exosomes leads to inconsistencies in their reported therapeutic effects [41,224]. This challenge is further complicated by the variability of exosome cargos, which includes therapeutic miRNAs and proteins, depending on both the cell passage number and the donor source. While the exosome cargos of OMSCs has not been directly tracked across passages, the parent MSCs are known to undergo phenotypic and genetic expression changes during long-term culture, which strongly suggests that the contents of their secreted exosomes also evolve over time [41,224,225]. Furthermore, there is clear evidence of donor-to-donor variability, as the originating microenvironment and specific tissue source are reflected in the exosome’s composition and function [41]. This inherent biological variability makes it difficult to compare results across studies and poses a significant hurdle for developing standardized, off-the-shelf exosome-based therapies.

#### 4.3.5. Study Design and Reporting

Many studies face challenges such as small sample sizes, limited follow-up periods, and potential publication bias, where positive results are more likely to be published, leading to an incomplete understanding of efficacy [2,202]. To address these inconsistencies, researchers have employed systematic review and meta-analysis methodologies to synthesize findings and quantify effect sizes, particularly for applications in osteoarthritis treatment and bone repair [26,164,226,227]. However, these analyses often conclude that substantial variability in study design, cell sourcing, and outcome reporting limits the ability to draw definitive conclusions, emphasizing the need for larger, standardized studies to facilitate more robust quantitative comparisons [2,41,93,165].

### 4.4. Limitations of Existing Technologies

The success of OMSCs therapy depends on the biomaterial scaffolds used for cell delivery. These scaffolds must have sufficient mechanical properties, enable controlled release of growth factors, replicate the natural bone ECM, and enhance vascularization; however, developing materials that fulfill all these requirements remains challenging [82]. A persistent difficulty lies in controlling the scaffold’s degradation rate to precisely match the pace of new tissue growth. This challenge is compounded by the vast differences in degradation profiles across available biomaterials. For example, synthetic polymers like PCL degrade slowly over years, while PLGA can be tuned to degrade over months, though its acidic byproducts can sometimes elicit an inflammatory response [39,82,228]. Natural polymers such as collagen may degrade too quickly without chemical cross-linking [229]. The resorption rate of bioceramics depends on the ratio of stable HA to the more resorbable β-TCP [6,230,231]. Consequently, no single standardized guidance exists for material selection. Instead, the choice must be tailored to the specific clinical application, balancing the need for initial mechanical stability with the requirement that the scaffold disappears as new, functional tissue forms. For example, one study using human dental pulp cells in a hyaluronic-based hydrogel scaffold observed incomplete bone regeneration where the scaffold was not fully degraded, resulting in entrapped cells and highlighting the importance of synchronized degradation [149]. Furthermore, the inherent complexity of craniofacial anatomy presents a high bar for success, requiring engineered structures that account for the specific size, shape, and specialized cellular composition of the target tissue while modulating blood supply and minimizing inflammatory responses [12,232].

While OMSCs present a promising avenue for craniofacial and other tissue regeneration due to their inherent qualities and accessibility, their journey toward widespread clinical adoption is constrained. Overcoming these challenges will require more standardized protocols for cell handling, a deeper understanding of their in vivo behavior in complex defects, and the continued development of advanced scaffold systems that can fully support and orchestrate their regenerative potential.

## 5. Enabling Future Technologies and Directions

### 5.1. Emerging Biological Applications

The frontiers for OMSC therapy are rapidly expanding into complex fields like neurodegeneration. Given their neural crest origin, OMSCs naturally secrete vital neurotrophic factors, including BDNF and NGF. This intrinsic property has positioned them as promising therapeutic candidates for conditions like Alzheimer’s and Parkinson’s disease, where they may help protect existing neurons and support endogenous nerve regeneration [198,233]. Beyond neurodegeneration, preliminary research is exploring the use of OMSCs for metabolic and retinal disorders. Studies are investigating whether the immunomodulatory properties of GMSCs can help preserve islet function in diabetes, or if certain OMSCs can be differentiated into insulin-producing cells. Similarly, their potential to regenerate retinal cells offers a theoretical avenue for treating diseases like age-related macular degeneration [220]. While many of these applications are still in early preclinical stages, they underscore the remarkable versatility and broad therapeutic potential of these easily accessible stem cells.

### 5.2. 3D Bioprinting and Advanced Scaffolds

The translation of regenerative therapies is increasingly reliant on advanced fabrication technologies that can construct functional, patient-specific tissues. Among these, three-dimensional (3D) bioprinting has emerged as a revolutionary approach, enabling the precise, layer-by-layer construction of scaffolds with architectures that mimic native tissue—a critical factor for successful integration, particularly for complex structures [112,234]. Based on computer-aided designs (CAD) derived from a patient’s medical imaging, this technology allows for the creation of customized, anatomically accurate constructs with controlled internal porosity, which is essential for cell infiltration and nutrient diffusion [92,101]. The versatility of 3D printing is reflected in its diverse techniques, from acellular methods like Stereolithography (SLA) and Selective Laser Sintering (SLS) that build the foundational scaffold, to bioprinting processes like Inkjet, Laser-Assisted (LAB), and Extrusion bioprinting, which incorporate living cells directly into a printable “bio-ink” [15,112,219].

The success of these techniques hinges on the development of advanced scaffold materials that are biocompatible, possess appropriate mechanical properties, and biodegrade at a rate that matches new tissue formation [92,101,112,235]. A wide array of materials is employed, including natural polymers like collagen and alginate, synthetic polymers such as Poly-Lactic Acid (PLA) and PCL, and osteoconductive ceramics like HA and β-tricalcium phosphate (β-TCP). Often, composite materials that combine the biological advantages of natural polymers or ceramics with the mechanical strength of synthetic polymers are used to achieve superior performance [86,236]. These materials can be fabricated into various functional forms to suit the specific clinical need, including porous scaffolds that guide tissue ingrowth, injectable hydrogels that fill irregular defects, and multi-layered cell sheets for complex tissue interfaces [9,12,39,50,70,92,109,112,237].

These advanced scaffolds are extensively explored for regenerating orofacial bone defects resulting from trauma, tumor resection, or congenital malformations [71,72,82,238]. In rat critical-size calvarial defects, for instance, scaffolds made of dense collagen gel seeded with DPSCs or 3D-printed poly(lactide) loaded with GMSCs have been shown to significantly enhance bone regeneration and vascularization [71,239]. For restoring mandibular defects, human alveolar bone-derived MSCs combined with porous nano-HA/collagen/PLA scaffolds have proven effective in rabbit models, while BFPDs cultured on bioceramics have improved bone healing in both healthy and osteoporotic rats [20,203]. This technology is also providing new hope for treating congenital alveolar clefts, offering an alternative to traditional autologous bone grafts by using MSCs mixed with PRF or delivering potent growth factors like rhBMP-2 on absorbable carriers to effectively repair the defect [77,232,240,241]. Furthermore, these strategies support dental applications by improving osseointegration, where MSCs and PRF combined with porous HA scaffolds have led to higher bone density around implants [6,67,232].

Beyond bone, 3D printing is being leveraged for orofacial soft tissue regeneration, overcoming the donor site morbidity and limited tissue availability associated with autologous grafts [71,82]. A major goal is the fabrication of patient-tailored gingival equivalents that recreate the full-thickness oral mucosa, for which GMSCs are an ideal cell source due to their ability to support regeneration and re-innervation [219]. An even greater challenge is the regeneration of the entire periodontal complex, a hierarchical structure of gingiva, periodontal ligament (PDL), cementum, and alveolar bone. Here, “cell sheet technology” using PDLSCs and 3D-printed multiphasic scaffolds is being developed to recapitulate this structural integrity [219]. By incorporating different materials (e.g., PCL/hydroxyapatite) and designs for the bone and PDL components, these advanced constructs can orchestrate the regeneration of the complete tooth-supporting apparatus [39,219].

The significant advantages driving the adoption of these fabrication methods are manifold [82,92,112,236]. They enable the creation of personalized scaffolds that perfectly fit a defect, and they offer precise architectural control over pore size and interconnectivity, which is crucial for vascularization and cell migration [92,235]. They also provide a platform to combine different materials, cells, and bioactive molecules in a spatially controlled manner to better mimic native tissue and enhance regeneration, thereby overcoming the limitations of traditional grafts [82,219,237]. However, while 3D printing and advanced scaffolds show immense promise, achieving anatomical structure does not always guarantee physiological function [15]. A primary challenge remains the creation of functional vascular networks within large constructs to ensure nutrient supply and cell survival [8,83]. Overcoming this hurdle will be critical for translating these powerful technologies from the bench to the clinic [232].

### 5.3. The Role of Artificial Intelligence in Regenerative Medicine

Artificial Intelligence (AI) is revolutionizing regenerative medicine by transforming the field from a trial-and-error process to a predictive, data-driven paradigm [101,242,243]. A primary application is the optimization of scaffold design, where AI models analyze complex relationships between biomaterial properties and cellular responses to predict architectures that enhance cell attachment, proliferation, and osteogenic differentiation—a critical step for therapies utilizing OMSCs [242]. Furthermore, when integrated with in vitro platforms like bone-on-a-chip (BOC) systems, AI facilitates high-throughput screening of therapeutic agents and analysis of OMSCs behavior, such as differentiation and cell–cell interactions, within tissue-mimicking microenvironments [17,20,197,244,245,246,247]. AI can also optimize the complex microenvironmental factors within these microfluidic devices, such as flow rates and mechanical stimuli, to better direct OMSCs functions [248,249,250].

At the molecular level, AI accelerates the understanding of disease pathophysiology and streamlines the discovery of bioactive molecules that enhance tissue regeneration [197]. A significant breakthrough is DeepMind’s AlphaFold model, which provides unprecedented accuracy in predicting the 3D structure of proteins from their amino acid sequence [244,251]. This capability is foundational for elucidating the structural basis of interactions between growth factors (e.g., VEGF, BMP) and their receptors, thereby deciphering the complex signaling cascades that govern bone healing [6,252]. For instance, AlphaFold2 was used to predict peptide-receptor binding, leading to the development of a peptide that enhanced the therapeutic potential of MSCs for bone regeneration [244]. Looking forward, AI holds immense promise for personalized medicine by analyzing patient-specific data to select optimal OMSCs sources and predict therapeutic responses [242]. While challenges such as the need for large, high-quality datasets remain, the synergy of AI with nanotechnology, genome editing, and bioprinting is poised to deliver novel, patient-specific therapies for bone regeneration [242].

### 5.4. Microfluidic Systems in Bone Tissue Engineering

Complementing the computational power of AI, microfluidics offers a powerful platform for stem cell research by reconstructing complex physiological microenvironments in vitro [242,246,253,254]. This technology enables the development of BOC models, which are advanced systems that recapitulate the native bone microenvironment and serve as robust alternatives to preclinical animal models [197,246,254,255,256,257]. Key features of these systems include the ability to engineer biomimetic architectures, apply controlled mechanical forces like fluid shear stress and compression crucial for osteogenesis, and support the stable co-culture of multiple cell types to study the intricate interactions governing bone remodeling and regeneration [197,253,254,255,258,259].

Although many foundational studies use other MSCs sources, the principles are directly applicable to OMSCs [65,177,260]. Microfluidic platforms are instrumental in designing therapeutic strategies, from fabricating advanced biomaterial scaffolds to directing the osteogenic differentiation of stem cells for cell-based therapies [111,245,246,249,261]. Furthermore, their small scale makes them ideal for the high-throughput screening of drugs and optimizing growth factor concentrations [197,213,246,250]. Despite challenges in fully replicating the complex bone matrix and overcoming material limitations, the future lies in integrating microfluidics with 3D bioprinting and AI-driven analysis to create sophisticated, personalized models and translate these platforms into point-of-care tools [111,213,254,259,261,262].

### 5.5. Cell-Free Therapies: The Potential of Exosomes

Exosome therapy is increasingly positioned not merely as an adjunct but as a promising cell-free alternative that could circumvent many challenges associated with direct cell-based therapies. Direct application of MSCs in translational medicine is hindered by critical issues such as insufficient tissue penetration, rapid clearance, immunological and mutagenic risks, and post-treatment complications like thromboembolism and fibrosis [41]. Exosomes are considered a “promising cell-free approach for regenerative therapy” because their stable properties and acellular nature may bypass these safety and logistical hurdles [13,165,263]. By harnessing the therapeutic cargo of their parent cells without the risks of administering the cells themselves, exosomes represent a significant strategic shift in regenerative medicine.

### 5.6. Strategies for Clinical-Scale OMSCs Production

A significant barrier to the clinical translation of OMSCs therapies is the challenge of producing sufficient quantities of high-quality, standardized cells. To address this, several scale-up strategies are being explored. To overcome the limited quantities of primary cells, generating OMSCs mimics from induced Pluripotent Stem Cells (iPSCs) is presented as a highly promising strategy. The controlled induction of iPSCs, for instance, from abundant and accessible human gingival fibroblasts, offers a method to obtain large, potentially patient-specific cell populations, bypassing the sourcing limitations of primary tissues [196,212,232].

Here, the transition from traditional 2D culture to 3D aggregate or spheroid culture is favored. This method allows for tight cell–cell and cell–matrix interactions that better mimic the native microenvironment, which has been shown to enhance the stemness, multipotency, and secretome profiles of MSCs while improving their resistance to oxidative stress [13,181]. Finally, advanced culture platforms like bioreactors and microfluidic systems provide the controlled environment necessary to optimize and standardize both iPSC differentiation and 3D aggregate formation at scale, representing a key enabling technology for manufacturing clinical-grade cell products.

### 5.7. Designing Multicenter Studies for Clinical Translation

Addressing the challenges of heterogeneity to meet regulatory standards for translation requires a multi-pronged strategy for designing future multicenter studies. A foundational element is the strict adherence to standardized protocols guided by Good Manufacturing Practices (GMP) [25,93]. This includes uniform, serum-free culture conditions; consistent methods for cell isolation and expansion; and robust characterization of cell products according to established criteria, such as those from the International Society for Cellular Therapy (ISCT) [7,225].

To generate more predictive data and bridge the gap between preclinical models and human outcomes, multicenter studies should leverage advanced platforms like organ-on-a-chip systems [190,255,256]. When integrated with biosensors and artificial intelligence, these models can better predict OMSCs behavior and therapeutic responses, thereby refining study design and identifying patient populations most likely to benefit [242,243,258]. Ultimately, generating the high-quality evidence demanded by regulatory bodies will depend on executing large-scale, randomized controlled clinical trials with long-term follow-up to definitively establish safety and efficacy [57].

The conceptual roadmap of OMSCs consists of five key components: cell source, scaffold design, delivery method, molecular cues, and clinical indications. The optimal combination of these elements leads to the successful regeneration of tissue. One of the most transformative contributions in orofacial stem cell biology is the induction of pluripotency through the ectopic expression of pluripotency-associated transcription factors. The immunomodulatory property of these cells would eliminate the use of autologous stem cells. Presumably, reprogramming stem cells is a functional equivalent of Embryonic Stem Cells. The selection of the scaffold and scaffold design is given the highest priority. Scaffolds should mimic the natural extracellular matrix, providing a structural and functional framework, as well as sustained delivery of biological cues. Taken together, the optimal combination of these factors contributes to successful tissue regeneration.

## 6. Conclusions

The field of regenerative medicine is at a pivotal juncture, poised between ground-breaking scientific discovery and the complex realities of clinical translation. Significant advancements have been made in identifying diverse stem cell populations, particularly OMSCs, which offer immense potential due to their neural crest origin and multipotent capabilities. The development of sophisticated biomaterials, notably 3D-printed wavy structures, has demonstrated an ability to directly influence cellular behavior and enhance regeneration. Moreover, the emergence of Organ-on-a-Chip (OOC) technology is revolutionary, providing physiologically relevant microenvironments that bridge the gap between simplified 2D cultures and complex animal models. The integration of AI is a transformative force, enabling researchers to analyze vast datasets, derive mechanistic insights, and design more effective therapeutic strategies.

However, the field is simultaneously grappling with persistent, formidable challenges. The “translational gap” remains a chasm between highly promising preclinical results and consistent, safe, and effective clinical applications. This is largely due to a pervasive lack of standardization across virtually all aspects of research—from cell isolation and expansion to scaffold properties and exosome preparation. This inherent variability makes reproducibility a significant hurdle. Furthermore, the precise mechanisms of action of many stem cell therapies are still not fully elucidated. Safety concerns, particularly regarding potential tumorigenicity of highly proliferative cells like iPSCs, continue to necessitate rigorous screening and regulatory oversight, while cost and scalability remain significant barriers.

The future of regenerative medicine lies in deepened interdisciplinary collaboration that integrates cell biology, bioengineering, computational science, and clinical practice. We must move beyond simply demonstrating “proof of concept” and focus on developing more predictive preclinical models that better mimic human conditions, including patient age and comorbidities. The industry needs to collectively establish robust, standardized, and globally recognized protocols for manufacturing and quality control of stem cell products to ensure safety and reproducibility.

The path forward requires bold investment in translational research, favoring randomized controlled clinical trials with long-term follow-ups to generate high-quality evidence. We must leverage AI not just for data analysis, but for the predictive design of new biomaterials and cell-based strategies, minimizing costly trial-and-error. The ability to personalize treatments, using patient-derived iPSCs in OOC models for drug screening, offers a glimpse into a future of precision medicine. In essence, the field is like a promising sapling with deep roots in scientific discovery and rapidly growing branches of technological innovation.

## Figures and Tables

**Table 1:** Comparative landscape of dental and craniofacial stem cells.

Cell Type	Markers and Proliferation *	Primary Differentiation Potential	Accessibility	Immunomodulatory Effects
**Dental Pulp Stem Cells (DPSCs)**	Standard MSC markers. High; faster rate than BMSCs [23].	Osteogenic: High potential for producing reparative dentin; sometimes superior to BMSCs [24,25]. Chondrogenic: Consistent differentiation [26]. Neurogenic: Differentiates into neuron-like, dopaminergic, and retinal ganglion cells [26].	Readily accessible from the neurovascular bundle of healthy, inflamed, or wisdom tooth pulp. Can be recovered from cryopreservation [12,24,27].	Immunologically privileged. Higher capacity than BMSCs; suppresses T-cell alloreactivity. Exosomes enhance secretion of anti-inflammatory IL-10 and TGF-β [28,29].
**Stem cells from exfoliated deciduous teeth (SHED)**	Express neural markers (nestin, βIII-tubulin, GFAP, NFM) in addition to standard MSC markers [30]. High proliferation rates, significantly greater than DPSCs [31].	Osteogenic: Can induce host cells to form bone and dentin [24,30]. Chondrogenic: Differentiates into chondrocytes [26]. Neurogenic: Differentiates into neuron-like cells, oligodendrocytes, adipocytes, and odontoblasts [16,32,33].	Obtained from naturally exfoliated deciduous (“baby”) teeth; considered disposable tissue with limited ethical concerns and no donor site morbidity [34,35].	Significant potential; adjusts CD4^+^ T-cell responses [36]. Neuroprotective effects. Conditioned medium (CM) and exosomes show potent anti-apoptotic and anti-inflammatory effects [37].
**Periodontal Ligament Stem Cells (PDLSCs)**	Standard MSC markers. High expansion capability; higher proliferation rate than DPSCs [38].	Osteogenic: Differentiates into osteogenic cells [39]. Chondrogenic: Potential is noted [24]. Neurogenic: Can transdifferentiate into retinal ganglion-like cells. Useful for tendon tissue regeneration [16,39].	Easily accessible from periodontal ligament of impacted third molars. Can be isolated from cryopreserved ligaments [22,24].	Properties similar to BMSCs; can suppress immune/inflammatory reactions [40]. Exosomes induce osteogenic differentiation and suppress macrophage inflammasome activation [41].
**Gingival Mesenchymal Stem Cells (GMSCs)**	Standard MSC markers. Homogenous population that proliferates faster than BMSCs [12].	Osteogenic: Forms calcified bone nodule structures. Differentiates into chondrocytes, adipocytes, and osteocytes [42]. Chondrogenic: Confirmed differentiation capacity [42]. Neurogenic: Aggregates into neurospheres with enhanced neuronal differentiation [43].	Easily accessible from gingival tissue (lamina propria) with minimal morbidity, rapid healing, and no scarring [12].	Potent immunomodulatory and anti-inflammatory functions, maintaining potential even in inflammatory environments [42]. Suppresses M1 macrophages while boosting M2. Promotes Regulatory T-cell (Treg) and suppresses lymphocyte proliferation [42,44].
**Dental Follicle Stem Cells (DFSCs)**	Express high levels of osteogenic markers (RUNX2, ALP) in addition to standard MSC markers [45].	Osteogenic: Higher potential than DPSCs and PDLSCs, particularly in inflammatory models [46]. Differentiates into cementoblasts and PDL fibroblasts [47]. Chondrogenic: Confirmed differentiation [48]. Neurogenic: Confirmed differentiation [49].	Easily isolated from the dental follicle of developing teeth (e.g., wisdom teeth). Highly plastic if sourced early [48,50].	Superior immune-suppressive effect compared to other dental MSCs [51]. Suppresses lymphocyte proliferation and increases Treg ratio, particularly in autoimmune contexts (e.g., RA) [51,52].
**Alveolar Bone-Derived Mesenchymal Stem Cells (ABMSCs)**	Implied standard MSC markers (as a BMSCs source).	Osteogenic: Confirmed. Effective for treating craniofacial defects and restoring alveolar bone voids [20]. Chondrogenic: Confirmed, but poor [53]. Neurogenic: Not specified, but neural crest origin suggests potential.	Sourced from alveolar bone marrow, often via minimally invasive aspirates during dental surgeries [53].	observed to be similar to those of standard BMSCs [54].
**Buccal Fat Pad-Derived Stem Cells (BFPDs)**	Implied standard MSC markers (as an adipose-derived source) [5,55].	Osteogenic: Confirmed [53]. Chondrogenic: Higher ability for transformation than GMSCs. Neurogenic: Not specified.	Available from buccal fat pad; facilitated preparation and lower morbidity than BMSCs harvest [17,56].	Not detailed, but shares similarities with immunomodulatory Adipose tissue-Derived Stem Cells (ADSCs) [56].
**Salivary Gland Stem Cells (SGSCs)**	Markers for source cells (e.g., EpCAM^+^, CD24^+^/CD29^+^), not traditional MSCs. Exhibit markers of both embryonic and adult stem cells [11,57]. Can proliferate and form organoids.	Can be induced to differentiate into chondrogenic, osteogenic, and adipogenic cells [58].	Isolated from salivary glands; focus is often on endogenous cellular plasticity rather than routine harvesting [57,59].	Indirectly contributes through secretion of paracrine factors (cytokines, growth factors, EVs) [60].
**Stem Cells from Apical Papilla (SCAPs)**	Standard MSC markers. Highly proliferative, with greater capacity than DPSCs [61].	Osteogenic: Differentiates into osteoblasts. Shows high in vitro mineralization potential [62]. Chondrogenic: Capable of cartilage differentiation [63]. Neurogenic: Not specified [64,65].	Isolated from the apical papilla of incompletely developed teeth. Easy to access [61].	Reported to have immunomodulatory characteristics similar to BMSCs [11,66].

* All cells mentioned typically show expression of standard MSC markers (CD73, CD90, CD105) and do not express hematopoietic markers (CD34, CD45).

**Table 2:** Key mediators of angiogenesis and osteogenesis in bone repair.

Mediator	Role in Angiogenic-Osteogenic Coupling
**Fibroblast growth factor (FGF)**	Particularly FGF-2, these are potent mitogens for mesenchymal cells and osteoblasts and are strongly angiogenic [113,114,115].
**Platelet-derived growth factors (PDGF)**	Released from platelets during clotting, PDGF is chemotactic for osteoblasts and promotes angiogenesis, partly by upregulating VEGF [116,117,118,119].
**Bone morphogenetic proteins (BMPs)**	Powerful osteoinductive factors (e.g., BMP-2, BMP-7) that stimulate osteoprogenitor differentiation. Their angiogenic effects are primarily indirect, mediated through the upregulation of VEGF [107,120,121].
**Placental Growth Factor (PlGF)**	A VEGF homolog that stimulates MSC proliferation and differentiation toward osteoblastic and osteoclastic lineages [99,122,123].
**Other Factors**	Molecules such as Insulin-like Growth Factor (IGF), Erythropoietin (EPO), and Angiopoietins (Ang-1, Ang-2) contribute to neovascularization and vessel maturation [99,110,116,124,125,126,127].
**Metallic Ions & Exosomes**	Emerging evidence shows that metallic ions (e.g., Co, Mg, Zn) can promote angiogenesis by upregulating HIF-1α and VEGF. Similarly, MSC-derived exosomes promote both angiogenesis and osteogenesis by activating pathways such as HIF-1α/VEGF and BMP-2/Smad1/Runx2 [100,128,129,130,131,132].

**Table 3:** Comparison of OMSC-based preclinical bone regeneration strategies.

Parameter	Mandiblar Defect Study [87,143,144]	Maxillary Alveolar Defect Study [18,142,145]	Calvarial Defect Study [71,146,147]
Stem cell sources	**Yamada et al. [87]:** Canine BMSCs (cMSCs), canine DPSCs (cDPSCs), and puppy deciduous teeth stem cells (pDTSCs). **Wang et al. [143]:** GMSCs. **Khosronejad et al. [148]:** DPSCs	**Kandalam et al. [18]:** GMSCs **Jahanbin et al. [142]:** DPSCs. **Seo et al. [145]:** SHED.	**Chamieh et al. [71]:** DPSCs. **Rezai-Rad et al. [146]:** Dental Follicle Stem Cells (DFSCs). **Johnson et al. [147]:** Dental pulp neural crest MSCs (DPNCCs) and bone marrow aspirate (BMA).
Scafold	**[87]:** PRP. **[143]:** Type I collagen gel. **[148]:** Composite scaffold. (PLA-HA-GLA) consisting of polylactic acid (PLA), HA nanoparticles (n-HA), gelatin, and hesperidin.	**[18]:** Hydrogel scaffold with PuraMatrix™ (PM) and/or BMP-2. **[142]:** Collagen membrane. **[145]:** HA/TCP.	**[71]:** Dense collagen gel. **[146]:** PCL. **[147]:** 3D printed osteoconductive HA/TCP.
Animal	**[87]:** Canine. **[143]:** Rat. **[148]:** Rat.	**[18]:** Rat. **[142]:** Rat. **[145]:** Mice.	**[71]:** Rat. **[146]:** Rat. **[147]:** Swine.
Conclusion	**[87]:** Both DPSCs and DTSCs yielded good bone formation. **[143]:** Repaired the defect. **[148]:** Repaired the defect.	**[18]:** PM with BMP-2 yielded best regeneration. **[142]:** Repaired the defect. **[145]:** Repaired the defects but failed to recruit hematopoietic marrow.	**[71]:** Bone mineral density increased with DPSC-seeded scaffolds. **[146]:** Bone regeneration was only observed in the defects treated with DFSC. **[147]:** Both repaired the defect.

**Table 4:** Clinical trial landscape.

Registration ID	Status (Phase)	Condition Investigated	Cell Type Used
NCT03386877 [204]	Completed (2017)	Periodontal Intrabony Defects	Autologous DPSCs
NCT03194451 [206]	Completed (2019)	Bone Tissue Engineering	Autologous DPSCs
NCT00221130 [207]	Completed (2005)	Adult Periodontitis	Autologous MSCs/Osteoblasts
NCT01357785 [208]	Completed (2014)	Periodontal Intrabony Defects	Autologous DPSCs
NCT01082822 [209]	Completed (2012)	Chronic Periodontitis	Autologous DPSCs
NCT04641533 [210]	Completed (2020)	Third Molar Extraction Sockets	Autologous DPSCs
NCT04608838 [205]	Completed (2021)	Acute Ischemic Stroke	Allogeneic DPSCs (JTR-161)
NCT03658655 [211]	Completed (2019)	Type 2 Diabetes	SHED

## Data Availability

Not applicable.
